# BRAF-activated long non-coding RNA contributes to colorectal cancer migration by inducing epithelial-mesenchymal transition

**DOI:** 10.3892/ol.2014.2154

**Published:** 2014-05-19

**Authors:** QINHAO GUO, YAN ZHAO, JIEJING CHEN, JUN HU, SHUWEI WANG, DONGSHENG ZHANG, YUEMING SUN

**Affiliations:** Department of Colorectal Surgery, The First Affiliated Hospital of Nanjing Medical University, Nanjing, Jiangsu 210029, P.R. China

**Keywords:** extracellular signal-regulated kinase, colorectal cancer, long non-coding RNA, BRAF-activated long non-coding RNA, migration, epithelial-mesenchymal transition

## Abstract

Long non-coding RNAs (lncRNAs) are recently identified regulators in tumorigenesis and tumour progression. BRAF-activated lncRNA (BANCR) is overexpressed in melanoma and has a potential functional role in melanoma cell migration. However, little is known concerning the role of BANCR in the development of colorectal cancer (CRC). The current study examined BANCR expression in 60 pairs of CRC and matched adjacent normal tissues. The quantitative polymerase chain reaction results showed that BANCR was frequently overexpressed in cancer tissues and this overexpression was found to significantly correlate with lymph node metastasis and tumour stage. The ectopic expression of BANCR contributed to the migration of human CRC Caco-2 cells, whereas knockdown of BANCR inhibited the migration of the HCT116 cells *in vitro*. Further investigation into the underlying mechanisms responsible for the migratory effects revealed that BANCR induced the epithelial-mesenchymal transition (EMT) through an MEK/extracellular signal-regulated kinase-dependent mechanism as treatment with the MEK inhibitor, U0126 decreased migration and reversed the EMT in the BANCR-overexpressed HCT116 cells. These results revealed the significance of BANCR in the molecular etiology of CRC and implied the potential application of BANCR in the therapeutic treatment of CRC.

## Introduction

Colorectal cancer (CRC) is one of the most common types of lethal cancer worldwide, with over a million new cases diagnosed annually (1). In China, the majority of patients with CRC are diagnosed at a late stage, however, early detection of the disease significantly enhances the probability of survival. Understanding of the mechanisms that result in CRC and the specific biomarkers for CRC progression must be enhanced to aid the prediction and improvement of clinical outcomes.

Long non-coding RNAs (lncRNAs) are >200 nucleotides and do not code for proteins, but interact with proteins (2,3). Although lncRNAs are not as well characterised as small non-coding (nc) microRNAs, lncRNAs are critical in the regulation of diverse cellular processes, such as stem cell pluripotency, cell growth, cell cycle, apoptosis, metabolism and cancer migration (4–10). Functional lncRNAs may be used for cancer diagnosis and prognosis, as well as serve as potential therapeutic targets. Functional lncRNAs are considered to be particularly promising candidates for future cancer diagnosis and therapeutic strategies (11). BRAF-activated lncRNA (BANCR) is a recurrently overexpressed, previously unannotated 693-bp transcript on chromosome 9 with a potential functional role in melanoma cell migration (12,13). BANCR is closely associated with ^V600E^BRAF, the most frequent mutation type of the BRAF gene. Furthermore, high frequencies of ^V600E^BRAF mutations are detected in malignant melanoma (70%), papillary thyroid cancer (36–53%), and CRC (5–22%) (14). A close correlation exists between the presence of ^V600E^BRAF and progression to the advanced stages of CRC (15), however, the expression pattern and biological functions of BANCR in CRC remain unclear.

The epithelial-mesenchymal transition (EMT) is a key step toward cancer invasion and metastasis. From a molecular perspective, EMT is characterised by the loss of epithelial markers, including E-cadherin and cytokeratins, and the upregulation of mesenchymal markers, such as N-cadherin and vimentin (16). The overexpression of ^V600E^BRAF is susceptible to transforming growth factor-β-induced EMT through an extracellular signal-regulated kinase (ERK)-dependent mechanism (17).

The aim of the present study was to detect the expression levels of BANCR in CRC, and to reveal the function and molecular mechanisms of BANCR in CRC.

## Materials and methods

### Tissue samples and cell culture

In total, 60 specimens of human CRC tissues and adjacent normal tissues were obtained between March 2012 and June 2013 from the First Affiliated Hospital of Nanjing Medical University, (Nanjing, China) with written informed consent obtained from all patients. The study was approved by the Protection of Human Ethics Committee of the First Affiliated Hospital of Nanjing Medical University. The diagnosis of CRC was histopathologically confirmed and no patient received preoperative treatment. The resected tissue samples were immediately frozen in liquid nitrogen and stored at −80°C until RNA extraction. Of the 60 patients, 27 exhibited lymph node (LN) metastasis, while 33 did not. The data collected from all subjects included age, gender, and CRC features, such as tumour size, location, histological grade, depth of invasion and serum carcinoembryonic antigen (CEA) value. The clinical stage of CRC was evaluated based on the tumour, node, metastasis (TNM) classification system (National Comprehensive Cancer Network 2012) (18). The human CRC cells were obtained from the Type Culture Collection of the Chinese Academy of Sciences (Shanghai, China) and preserved at the First Affiliated Hospital of Nanjing Medical University. All cells were maintained in the recommended culture conditions and incubated at 37°C in a 5% CO_2_ humidified atmosphere.

### Quantitative polymerase chain reaction (qPCR)

Total RNAs from the tissues and cells were extracted using RNAiso Plus (Takara Bio, Inc., Dalian, China) and reverse transcription (RT) reactions were performed using a PrimeScript^TM^ RT reagent kit (Takara Bio, Inc.) according to the manufacturer’s instructions. For qPCR, a final volume of 20 μl reaction was performed according to a standard protocol and the SYBR Green PCR kit (Roche Diagnostics, Indianapolis, IN, USA) on the StepOnePlus Real-Time PCR System (Applied Biosystems, Carlsbad, CA, USA). The qPCR was performed in triplicate, with no template controls. The 2^−ΔΔCT^ method was conducted to determine the relative gene expression levels with β-actin serving as the endogenous control to normalise the data. The primers used were as follows: Forward, 5′-ACAGGACTCCATGGC AAACG-3′ and reverse, 5′-ATGAAGAAAGCCTGGTGC AGT-3′ for BANCR; forward, 5′-GTGTCATCCAACGGA ATGC-3′ and reverse, 5′-TGGCGGCATTGTAGGTGTTC-3′ for E-cadherin; forward, 5′-ATGACCGCTTCGCCAACTAC-3′ and reverse, 5′-CGGGCTTTGTCGTTGGTTAG-3′ for vimentin; and forward, 5′-AGA AAATCTGGCACCACACC-3′ and reverse, 5′-TAGCACAGCCTGGATAGCAA-3′ for β-actin. The PCR was performed using the following cycles: 95°C for 30 sec; 40 cycles of 95°C for 5 sec and 60°C for 31 sec; and a dissociation stage at 95°C for 15 sec, 60°C for 1 min and 95°C for 15 sec.

### Generation of stably infected cell lines

Recombinant lentiviruses containing short hairpin (sh)RNA-323 (LV-BANCR-323), shRNA-540 (LV-BANCR-540), human full-length BANCR cDNA (LV-BANCR) and negative control (LV-NC) were purchased from GenePharma (Shanghai, China). The HCT116 cells were infected with LV-BANCR-323, LV-BANCR-540 and LV-NC [multiplicity of infection (MOI)=20] and the Caco-2 cells were infected with LV-BANCR and LV-NC (MOI=10). The supernatant was removed after 24 h and replaced with a fresh culture medium. The infection efficiency was confirmed by qPCR 72 h following infection and the cells were treated with 2 μg/ml puromycin for two weeks.

### Wound healing experiments

The cells (5×10^5^) were seeded in 6-well plates and after 24 h, when the cells had grown by 90–100%, they were scraped with a pipette tip to generate straight wounds. To ensure documentation of the same region, the wells were marked across the wounded area. The medium was replaced with a serum-free medium (RPMI-1640, Wisent Inc., St-Bruno, QC, Canada) and the cells were treated with a medium containing 1 mM mitomycin to inhibit cell division. Phase-contrast images were recorded under an inverted microscope (Nikon ECLIPSE Ti-E, Nikon, Kobe, Japan) at the time of wounding, 0 h, and at 24 h. The untreated cells served as controls.

### Transwell migration assay

For the Transwell migration assays, 3×10^4^ HCT116 cells and 5×10^4^ Caco-2 cells were plated on the non-coated membrane on the top chamber (24-well insert; 8-mm pore size; Corning Costar Corp., Cambridge, MA, USA). The cells were plated on a medium without serum and a medium, which was supplemented with serum, served as a chemotactic agent in the lower chamber. The cells were incubated for 48 h and the cells that did not migrate through the pores were removed with a cotton swab, while cells on the lower surface of the membrane were stained with crystal violet (Beijing Solarbio Science & Technology Co., Ltd., Beijing, China). The cell numbers were determined by counting the penetrating cells under a microscope (Nikon ECLIPSE Ti-E) in random fields (five fields per chamber). Each experiment was performed in triplicate.

### Western blot analysis

Proteins were extracted with radioimmunoprecipitation assay (Beyotime, Shanghai, China) and equal amounts of protein were electrophoresed on a 6, 10 or 12% sodium dodecyl sulphate-polyacrylamide gel and subsequently transferred to polyvinylidene fluoride membranes (Millipore, Billerica, MA, USA). The membranes were blocked in 5% non-fat milk in Tris-buffered saline containing 0.1% Tween-20 (TBST) at room temperature for 2 h. The membranes were incubated overnight with the following primary antibodies at 4°C: E-cadherin (1:1,000; Cell Signalling Technology, BSN, USA), vimentin (1:1,000, Cell Signalling Technology) and GAPDH (1:10,000; Beijing Biosynthesis Biotechnology, Beijing, China). The membranes were washed thrice with TBST and incubated with horseradish peroxidase-conjugated secondary antibody (1:1,000; Beijing Biosynthesis Biotechnology) at room temperature for 2 h. Following three TBST washes, the membranes were developed using ECL Plus (Millipore, MA, USA) and exposed to X-ray film for visualisation of the protein bands. GAPDH served as an internal loading control.

### Statistical analysis

Data were analysed using SPSS 19.0 (IBM, Armonk, NY, USA) and were expressed as the mean ± standard error of mean. The significance of the differences between groups was estimated by Student’s t-test, Pearson’s χ^2^-test, or one-way analysis of variance, as appropriate. Two-sided P<0.05 was considered to indicate a statistically significant difference.

## Results

### BANCR expression in tissues and cell lines

**Figure 1:**
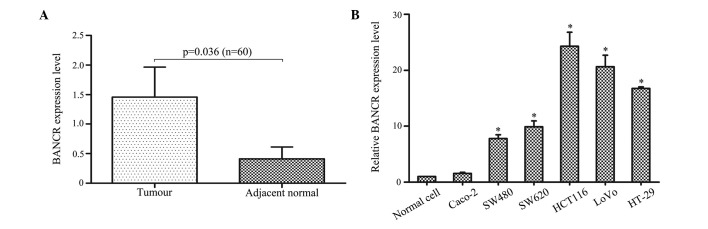
BANCR levels were upregulated in CRC. (A) BANCR expression levels assessed by qPCR in tumour tissues and adjacent normal tissues (n=60). BANCR expression levels were normalised to β-actin. (B) BANCR levels were determined by qPCR in six CRC cell lines (Caco-2, HCT 116, LoVo, SW480, SW620 and HT-29) and the CCD 841 CoN normal intestinal mucous cell line. Data are presented as the mean ± standard error of the mean. ^*^P<0.05. BANCR, BRAF-activated long non-coding RNA; CRC, colorectal cancer; qPCR, quantitative polymerase chain reaction. ^*^P<0.05 vs. normal cell.

The primary aim of the present study was to investigate whether BANCR was detectable and altered in 60 pairs of CRC tissues compared with adjacent normal tissues. The qPCR results demonstrated that BANCR expression was significantly higher in the tumour tissues than in the adjacent normal tissues (P<0.05; Fig. 1A). The qPCR assays were further developed to quantify the BANCR expression in the CRC cell lines. A higher BANCR expression was found in the HCT116 (P<0.01), LoVo (P<0.01), HT-29 (P<0.01), SW480 (P<0.05) and SW620 (P<0.05) cells when compared with the CCD 841 CoN normal intestinal mucous cell line, however, no significant difference was identified in the Caco-2 cells (P>0.05; Fig. 1B).

### Correlation between BANCR expression and the clinicopathological parameters of patients with CRC

The 60 patients with CRC were divided into high- (n=18) and low-BANCR expression (n=42) groups according to the mean value of the expression levels of BANCR in tumour tissues. Clinicopathological factors were analysed between the two groups and the high-BANCR expression group showed more advanced LN metastasis and tumour stage than the low-BANCR expression group (P<0.05). However, no significant correlation was found between the BANCR expression and other clinicopathological features, such as age, gender, tumour size, location, histological grade, depth of invasion and serum CEA value (P>0.05; Table I).

### Modulation of BANCR expression affects cell migration

**Figure 2:**
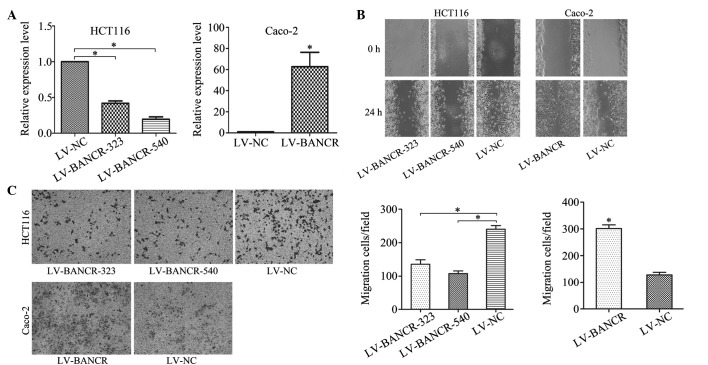
Modulation of BANCR expression affects cell migration. (A) Following treatment with LV-BANCR-323 and LV-BANCR-540, BANCR expression in HCT116 cell lines was downregulated compared with those treated with LV-NC, and BANCR expression in Caco-2 cell lines infected with LV-BANCR was significantly upregulated. (B) Wound healing experiments and (C) Transwell migration assays showed that BANCR overexpression promoted cell migration in Caco-2 cells, however, cell migration reduced in BANCR-downregulated HCT116 cells (crystal violet stain; magnification, ×100). The bar graph represent at least three independent experiments and the bars indicate the number of migrated cells per field. ^*^P<0.05. LV-BANCR-323, recombinant lentiviruses containing shRNA-323; LV-BANCR-540, recombinant lentiviruses containing shRNA-540; LV-BANCR, recombinant lentiviruses containing human full-length BANCR cDNA; NC, negative control; BANCR, BRAF-activated long non-coding RNA; shRNA, short hairpin RNA.

To clarify the role of BANCR within CRC cells, Caco-2 cells were transfected with LV-BANCR, which express relatively low levels of endogenous BANCR within CRC cell lines, while LV-BANCR-323 and LV-BANCR-540 were transfected with stable BANCR-expressing HCT116 cells. As a result, the BANCR expression was significantly downregulated in the HCT116 cells and upregulated in the Caco-2 cells (Fig. 2A). The wound healing experiment (Fig. 2B) and Transwell migration assay (Fig. 2C) showed that BANCR overexpression promoted cell migration in the Caco-2 cells, which was reduced in the BANCR-downregulated HCT116 cells.

### BANCR induces EMT phenotypes

**Figure 3:**
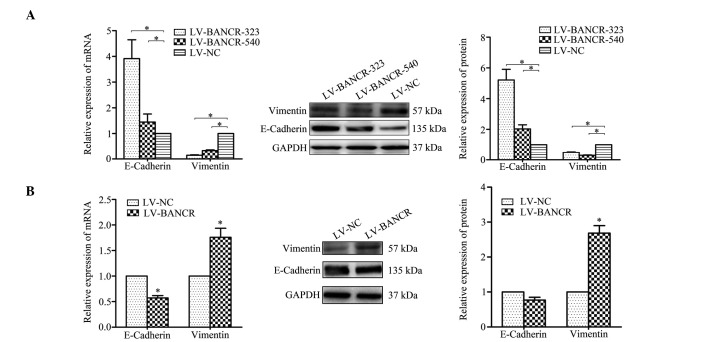
Modulation of BANCR expression affects the expression of E-cadherin and vimentin. (A) BANCR downregulation in HCT116 cells was associated with downregulated vimentin, and upregulated E-cadherin mRNA and protein levels. (B) BANCR overexpression in CACO-2 cells was associated with upregulated vimentin and downregulated E-cadherin. ^*^P<0.05. LV-BANCR-323, recombinant lentiviruses containing shRNA-323; LV-BANCR-540, recombinant lentiviruses containing shRNA-540; LV-BANCR, recombinant lentiviruses containing human full-length BANCR cDNA; NC, negative control; BANCR, BRAF-activated long non-coding RNA; shRNA, short hairpin RNA.

EMT is one of the key processes for primary tumour cells to acquire a migratory capacity (16). To define the role of BANCR in the progression of cell migration within CRC cells, the changes in expression of epithelial and mesenchymal markers were detected following modulation of the BANCR expression level. The downregulation of the BANCR levels within the HCT116 cells was found to be associated with the upregulated E-cadherin and downregulated vimentin expression at the mRNA and protein levels (Fig. 3A). Vimentin expression was upregulated and E-cadherin was downregulated in the BANCR-overexpressed Caco-2 cells (Fig. 3B).

### BANCR contributes to HCT116 cell migration through an ERK-dependent mechanism

**Figure 4:**
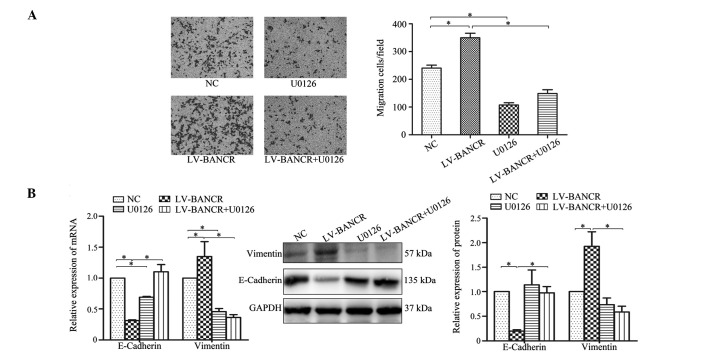
BANCR contributes to HCT116 cell migration via an extracellular signal-regulated kinase-dependent mechanism. (A) Transwell migration assay showed that treatment with MEK inhibitor, U0126, reduced HCT116 migration and that treatment with LV-BANCR induced HCT116 migration, however, this was also inhibited by U0126 (crystal violet stain; magnification, ×100). (B) Following treatment with U0126, LV-BANCR and LV-BANCR + U0126, the expression of E-cadherin and vimentin was impaired. ^*^P<0.05. LV-BANCR, recombinant lentiviruses containing human full-length BANCR cDNA; NC, negative control; BANCR, BRAF-activated long non-coding RNA; shRNA, short hairpin RNA.

Considering that the MEK signalling pathway has a specific link with the BRAF gene (19), the correlation between BANCR and the MEK signalling pathway was investigated and treatment with the specific MEK inhibitor, U0126 (30 μM) appeared to reduce HCT116 migration. Next, stable cell lines were generated that expressed BANCR cDNA in the HCT116 cells by lentiviral transfection with LV-BANCR. As predicted, LV-BANCR significantly increased the cell migration, however, the cell migration was decreased as a result of treatment with U0126 (Fig. 4A). In addition, BANCR overexpression was found to upregulate vimentin expression and downregulate E-cadherin expression (mRNA and protein). By contrast, treatment with LV-BANCR+U0126 was found to downregulate vimentin expression and upregulate E-cadherin expression (mRNA and protein; Fig. 4B). These results showed that BANCR contributes moderately to HCT116 cell migration via an ERK-dependent mechanism.

## Discussion

Advances in molecular techniques have led to the discovery of a novel type of regulatory gene. Although initially argued to be spurious transcriptional noise, lncRNAs are implicated to have oncogenic and tumour-suppressing roles (20,21). In CRC, one example of such oncogenic lncRNA is the Hox transcript antisense intergenic RNA (HOTAIR). HOTAIR expression levels are higher in cancerous tissues than in corresponding non-cancerous tissues of stage IV CRC patients. Furthermore, patients with high HOTAIR expression exhibit a relatively poorer prognosis (22). An additional classic oncogenic lncRNA is termed the highly upregulated in liver cancer (HULC). Colorectal carcinomas, which metastasise to the liver and not to the LNs, upregulate HULC ncRNA (23). MALAT-1, H19 and CRNDE RNAs are also associated with CRC (24–26).

BANCR is recurrently overexpressed in melanoma. The shRNA-mediated knockdown of BANCR in melanoma cells changes the expression levels of 88 genes, a number of which are involved in cell migration and chemotaxis. In addition, BANCR depletion impairs the migration of melanoma cells *in vitro* (12,13). In the present study, the BANCR expression levels in CRC patients were examined and the associated clinical implications were investigated. The qPCR showed that BANCR was upregulated in CRC tumour tissues compared with adjacent normal tissues. Furthermore, upregulated BANCR expression was found to correlate with the TNM stage and LN metastasis. In addition, the potential role of BANCR in CRC cells *in vitro* was examined. Altered BANCR expression in the HCT116 and Caco-2 cells was found to affect migration and thus, the current results are consistent with those of a previous study (13), indicating that BANCR expression enhances the aggressive biological behaviour of cancer cells of various origins. However, considering that the tissues were resected in the recent two years, the long-term overall survival rates were not analysed, however, will be investigated in future studies.

EMT processes, which are characterised by diminished epithelial characteristics and increased mesenchymal attributes, provide epithelial cells with enhanced migratory potential and are implicated in numerous physiological and pathological processes that require cell migration. LncRNA is important in regulating cancer progression (6,7) and therefore, recent studies are beginning to elucidate the association between EMT and lncRNAs. Ying *et al* (27) demonstrated that upregulated MALAT-1 contributes to bladder cancer cell migration by activating Wnt signalling and subsequently inducing EMT. Luo *et al* (28) observed that lncRNA H19 increases bladder cancer metastasis by associating with EZH2 and inhibiting E-cadherin expression. In addition, Xu *et a*l (29) showed that the knockdown of lncRNA HOTAIR suppresses tumour invasion and reverses EMT in gastric cancer. Although direct supporting evidence is insufficient, there is a suspected role of BANCR in the regulation of EMT. The present *in vitro* study identified that altered BANCR expression affects the expression of epithelial and mesenchymal markers, implying that BANCR may regulate EMT. Furthermore, the effect of BANCR on cell migration and EMT regulation may depend on the MEK/ERK signalling pathway as the MEK/ERK signalling pathway inhibitor, U0126, inhibits the BANCR overexpression-induced promotion of cell migration, vimentin upregulation and E-cadherin downregulation. These observations indicate that BANCR contributes to CRC migration by inducing EMT via an MEK/ERK-dependent mechanism.

In conclusion, to the best of our knowledge, the present study is the first to report that BANCR is highly expressed in CRC and that BANCR is likely to present as a useful biomarker of CRC. Furthermore, identifying that BANCR induces CRC migration by inducing EMT via an MEK/ERK signalling pathway expands on the current understanding of the molecular mechanisms of BANCR. Most importantly, BANCR may be used as a potential molecular target during the treatment of human CRC.

## Figures and Tables

**Table I:** Correlation between BANCR expression and clinicopathological parameters of colorectal cancer patients.

		BANCR expression				
			
Characteristic	n	High (n=18)	%	Low (n=42)	%	P-value
Age, years (mean ± SEM)	60	60.4±17.3		64.6±14.5		0.333
Gender						0.253
Male	35	13	37.1	22	62.9	
Female	25	5	20.0	20	80.0	
Tumour size, cm						0.253
<4	25	5	20.0	20	80.0	
≥4	35	13	37.1	22	62.9	
Location						0.167
Colon	32	7	21.9	25	78.1	
Rectum	28	11	39.3	17	60.7	
Histological grade						0.207
Well/moderately	44	11	25.0	33	75.0	
Poorly/other	16	7	43.8	9	56.3	
Depth of invasion						0.192
T1, T2	15	2	13.3	13	86.7	
T3, T4	45	16	35.6	29	64.4	
Lymph node metastasis						0.010^b^
Absent	33	4	12.1	26	78.8	
Present	27	14	51.9	16	59.3	
Serum CEA value, μg/l						0.085
<5	35	7	20.0	28	80.0	
≥5	25	11	44.0	14	56.0	
Tumour stage^a^						<0.001^b^
I, II	28	2	7.0	26	93.0	
III, IV	32	16	50.0	16	50.0	

a Tumour stage was obtained according to the tumour, node, metastasis criteria (National Comprehensive Cancer Network, 2012).

b P<0.05 vs. present and III, IV.

BANCR, BRAF-activated long non-coding RNA; CEA, carcinoembryonic antigen; SEM, standard error of the mean.
